# Induction of Mincle by *Helicobacter pylori* and consequent anti-inflammatory signaling denote a bacterial survival strategy

**DOI:** 10.1038/srep15049

**Published:** 2015-10-12

**Authors:** Savita Devi, Eerappa Rajakumara, Niyaz Ahmed

**Affiliations:** 1 Pathogen Biology Laboratory, Department of Biotechnology and Bioinformatics, University of Hyderabad, Hyderabad, Telangana, India; 2 Department of Biotechnology, Indian Institute of Technology Hyderabad, Yeddumailaram, Telangana, India

## Abstract

Evasion of innate immune recognition is one of the key strategies for persistence of *Helicobacter pylori*, by virtue of its ability to modulate or escape the host innate immune receptors and signaling pathways. C-type lectin receptors (CLRs) predominantly expressed by macrophages are pivotal in tailoring immune response against pathogens. The recognition of glyco or carbohydrate moieties by Mincle (Macrophage inducible C-type lectin) is emerging as a crucial element in anti-fungal and anti-mycobacterial immunity. Herein, we demonstrate the role of Mincle in modulation of innate immune response against *H. pylori* infection. Our results revealed an upregulated expression of Mincle which was independent of direct host cell contact. Upon computational modelling, Mincle was observed to interact with the Lewis antigens of *H. pylori* LPS and possibly activating an anti-inflammatory cytokine production, thereby maintaining a balance between pro- and anti-inflammatory cytokine production. Furthermore, siRNA mediated knockdown of Mincle in human macrophages resulted in up regulation of pro-inflammatory cytokines and consequent down regulation of anti-inflammatory cytokines. Collectively, our study demonstrates a novel mechanism employed by *H. pylori* to escape clearance by exploiting functional plasticity of Mincle to strike a balance between pro-and anti-inflammatory responses ensuring its persistence in the host.

*Helicobacter pylori* is a highly successful human pathogen that colonizes the human gastric mucosa of over half of the world’s population^1^. Although majority of colonized cases remain asymptomatic, infection with *H. pylori* leads to chronic inflammation in a fraction of colonized individuals and is the major cause of gastric cancer^2^,^3^,^4^. Apart from its association with cancer, *H. pylori* is different from other Gram-negative pathogens in its acumen to persist and establish chronic infection^5^.

The successful survival and persistence of *H. pylori* in human gut is achieved by multiple virulence factors such as CagA, VacA, HP0986, JHP0940, peptidyl propyl cis trans isomerase, OipA, GGT and DupA etc. which not only confer pathogenicity but also enable its persistent colonization^6^,^7^,^8^,^9^,^10^,^11^,^12^,^13^. In addition, *H. pylori* LPS also contributes in colonization by expressing the Lewis blood group antigens, Le^x^, Le^y^, Le^a^, Le^b^ in its fucosylated O-antigen; these Lewis antigens are also expressed by gastric epithelial cells resulting in a molecular mimicry^14^,^15^,^16^,^17^. This unique disposition of *H. pylori* with respect to its capsular composition contributes towards successful inhabitation and eventual long term interaction with its host^18^. LPS of *H. pylori* exhibits phase variation which attributes to induce immune tolerance by the bacterium^19^,^20^. Despite the availability of multiple virulence factors, long term colonization of *H. pylori* in human gut is also attributed to its ability to modify and subvert the activated innate immune response as well as adaptive immunity by modulation of effector T cell functions^5^. Innate immunity is the prerequisite for host defense mechanisms and is initiated by the recognition of “pathogen-associated molecular patterns (PAMPS)” *via* germ line encoded pattern recognition receptors (PRRs)^21^,^22^. Although PRRs are also expressed by gastric epithelial cells, macrophages act as the real mediators of inflammation to ward off the invading pathogens^23^,^24^,^25^. There are mainly four types of PRRs which comprise of Toll like receptors (TLRs), Nod Like receptors (NLRs), RIG like receptors (RLRs), and C-type lectin receptors (CLRs)^26^. CLRs are a diverse class of carbohydrate recognition receptors that are recently being studied for their crucial role in the recognition of microbial ligands including bacteria, fungi and viruses^27^,^28^. Macrophage-inducible C-type lectin (Mincle), which is also termed Clec4E and Clecsf9 is a key macrophage surface-expressed PRR. It is a 219aa, type II transmembrane protein with a carbohydrate recognition domain (CRD) in its extracellular region^29^.

Interest in Mincle as a significant mediator of diverse immune interactions has grown exponentially in recent years^28^. Various kinds of ligands specific to Mincle have been studied and these include SAP130 (SIN3A- associated protein) - a component of small nuclear ribonucleoprotein released from dead cells, polysaccharides that appear on bacterial and fungal surfaces of organisms, namely, *Mycobacteria* and *Candida* species and the components of recognition of self from non-self-antigens^30^,^31^,^32^. Such abundance of Mincle ligands points to its plausible interaction with other major pathogens such as *H. pylori*. However, characteristics of many such ligands concerning C-type lectin receptors on macrophages are still unknown^30^ and significance of any putative interactions involving Mincle are not fully understood at least in case of ligands emanating from Gram negative bacteria.

The progression of *H. pylori* induced lesions from superficial gastritis to chronic gastritis and eventually leading to gastric cancer could be reflective of sustained involvement of host inflammatory responses^33^,^34^. Consequently, the identification of factors that modulate host inflammatory responses may provide important insights about the mechanism of persistence and pathology. Though numerous prior studies have reported recognition of *H. pylori* at the level of TLRs and NLRs^35^,^36^,^37^, the role of CLRs, and Mincle in particular, remains underexplored.

Given this, we investigated the possible involvement of Mincle in recognition of *H. pylori.* Enhanced expression of Mincle was observed in human macrophages upon *H. pylori* infection. We also identified that Mincle acts as an important signalling receptor for *H. pylori* through both contact dependent and independent manner. Furthermore, by using Mincle knockdown macrophages, we demonstrate that Mincle mediated recognition of *H. pylori* influences both the pro- and anti-inflammatory cytokine responses. Collectively, our findings reveal that *H. pylori* exploits Mincle receptor to achieve a balance of pro- and anti-inflammatory responses and thus modulates the immune system in order to avoid clearance and gain persistence.

## Results

### Mincle expression elevated in *H. pylori* infected macrophages

**Figure 1:**
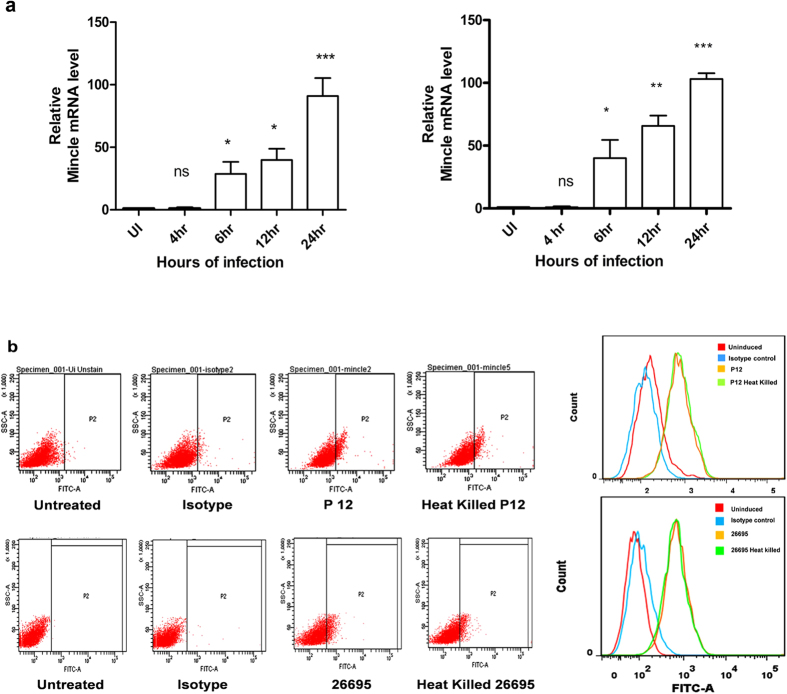
Mincle expression in THP-1 cells post infection with *H. pylori*. (**a**) Time course of Mincle mRNA expression was analysed by quantitative real time-PCR (qRT-PCR).THP-1 cells were infected with *H. pylori* strains P12 (left panel) and 26695 (right panel) at multiplicity of infection (MOI) of 50. Expression of Mincle was normalized with PPIB (peptidlypropylisomerase B) expression and is presented relative to expression in untreated THP-1 cells. Values are mean ± s.e.m. The data were analysed by 1 way ANOVA followed by Tukey’s multiple comparison test set as *P < 0.05, **P < 0.01, ***P < 0.001. (**b**) Surface expression of Mincle was analysed by flow cytometry in THP-1 cells post infection with *H. pylori* P12 and 26695 at 24 h. Infected THP-1 cells were stained with monoclonal anti-mincle antibody for Mincle expression and compared against un-infected cells (UI). Isotype antibody staining is represented in blue outline and uninfected/uninduced cells in red outline.

Activation of Mincle has been studied in response to *M. tuberculosis* and various fungal species^31^,^32^. We investigated whether Mincle expression was upregulated upon *H. pylori* infection as well. Mincle mRNA expression was quantified in THP-1 cell line in response to *H. pylori* infection *in vitro* by using qRT-PCR and was validated by melting curve. A time course analysis of Mincle expression levels were carried out from 4 h to 24 h post infection and we observed that Mincle mRNA transcript levels were increased in a time dependent manner. Our results demonstrated a significant increase in the transcript level of Mincle mRNA as early as 6 h post infection and increased up to 200 fold at 24 h post infection as compared to uninfected THP-1 cells. (Fig. 1a). This suggests that THP-1 cells sense *H. pylori* and upregulate Mincle receptor possibly for initiating the immune response against *H. pylori*. To further investigate the surface expression of Mincle upon *H. pylori* infection on THP-1 cells, flow cytometry was performed and our results indicate enhanced levels of Mincle expression on the surface of infected cells as compared with the non-infected cells (Fig. 1b). This indicates that *H. pylori* strongly interacts with Mincle and its expression on macrophages increases after *H. pylori* infection.

### Mincle expression is independent of live or heat killed *H. pylori*

**Figure 2:**
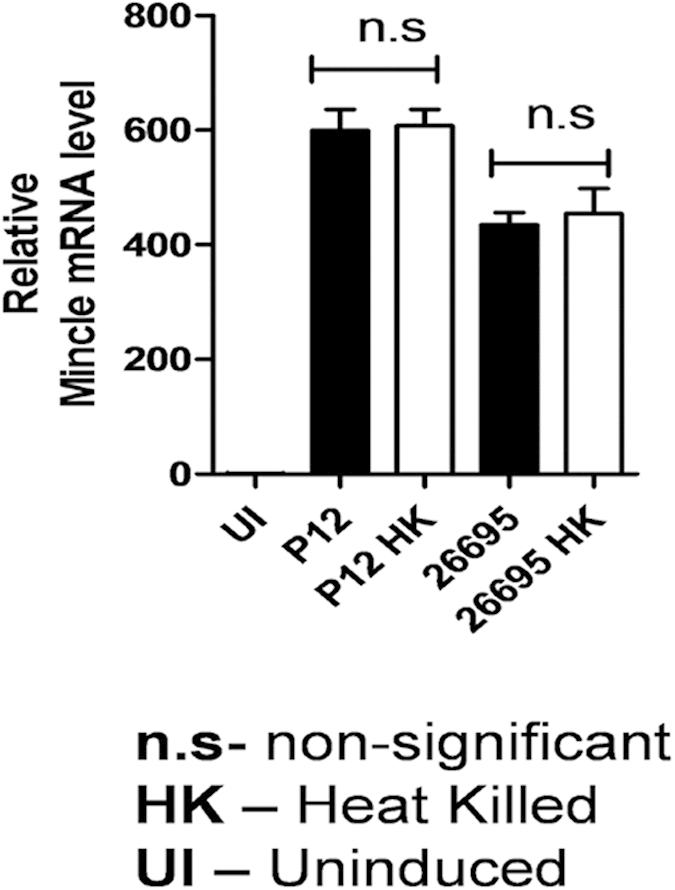
Mincle recognizes heat killed *H. pylori* strains. PMA differentiated THP-1 cells were co-cultured separately with live and heat killed *H. pylori* P12 and 26695 strains for 24 h and Mincle mRNA expression was quantified by qRT-PCR.*H. pylori* strains were heat killed at 98 °C for 20 min. Mincle mRNA expression was normalized with PPIB (peptidlypropylisomerase) mRNA expression. Mincle expression presented as relative to un-infected THP-1 cells. Data are mean ± s.e.m of three independent experiments, and were analysed by one way ANOVA followed by Tukey’s multiple comparison test.

We also investigated the capability of heat killed and live *H. pylori* strains to induce Mincle expression in THP-1 cells. Our results demonstrated that the heat killed *H. pylori* also upregulated Mincle expression with no significant difference (Fig. 2). As Mincle possesses a carbohydrate recognizing domain (CRD) in its extracellular region, it may recognize any specific carbohydrate structure of *H. pylori*. Above results also hint at the possibility that Mincle could be interacting with any non-protein entity of *H. pylori*.

### Mincle expression and cytokine secretion in THP-1 cells is independent of host cell contact

**Figure 3:**
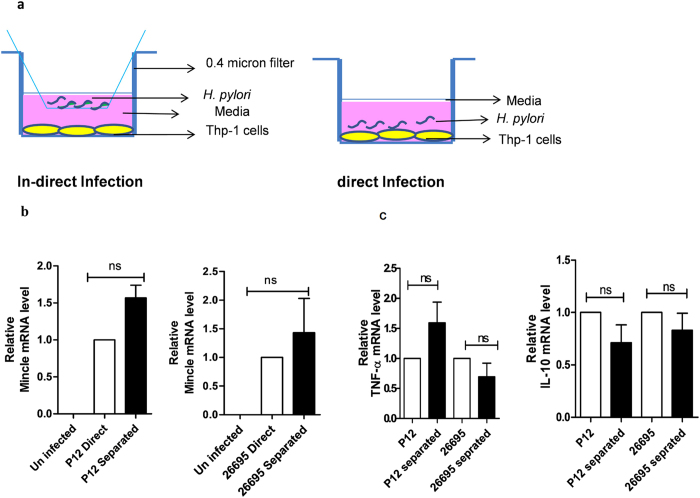
(**a**) Schematic representation of experimental design: PMA differentiated THP-1 cells were cultured at the bottom of the trans-well plate and *H. pylori* were separated from cells on the upper compartment having 0.4micron filter (In-direct infection).Uninfected control cells and cells infected directly with *H. pylori* (direct infection) were cultured normally in a 6 well plate. (**b**) Mincle expression is independent of THP-1 cells contact: Differentiated THP-1 cells were infected with *H. pylori* for 24 h and Mincle transcription was quantified by qRT-PCR. No significant fold change was observed in Mincle transcription level between directly infected THP-1 cells and indirectly infected THP-1 cells (separated). Results are presented relative to directly infected THP-1 cells and were normalized with PPIB housekeeping gene. Data are representative of three independent experiments (mean ± s.e.m) and analysed by student’s t test. (**c**) Quantitative RT-PCR analysis of TNF-a and IL-10 in THP-1 cells: TNF-a and IL-10 mRNA levels were quantified in both directly infected and indirectly infected THP-1 cells (separated) after 24 h. Mincle mRNA levels were normalized by PPIB as endogenous control and shown as fold change relative to directly infected THP-1 cells.

Further, we examined whether Mincle expression was host cell contact dependent or otherwise (Fig. 3a). qRT-PCR analysis revealed that there was no significant difference in Mincle mRNA expression levels in *H. pylori* separated THP-1 cells and cells directly in contact with *H. pylori* (Fig. 3b). In our observation, Mincle was expressed in a host cell contact independent manner. We also investigated if the expression of pro and anti-inflammatory cytokines might also be regulated at the level of host cell contact/ or independent manner during infection of THP-1 cells. To investigate this, a similar strategy was employed and the level of TNF-α and IL-10 production was estimated both at transcript and protein levels. Interestingly, no significant difference in the production of TNF-α and IL-10 was observed for both directly infected cells as well as in separated THP-1 cells (Fig. 3c). One possible explanation for such an observation could be due to the fact that most of the Gram negative bacteria continuously release LPS during growth or in stationary phase^38^ that might cross through the filters to induce expression of Mincle and inflammatory responses against *H. pylori*.

### *H. pylori* shed LPS during infection with THP-1 cells

**Figure 4:**
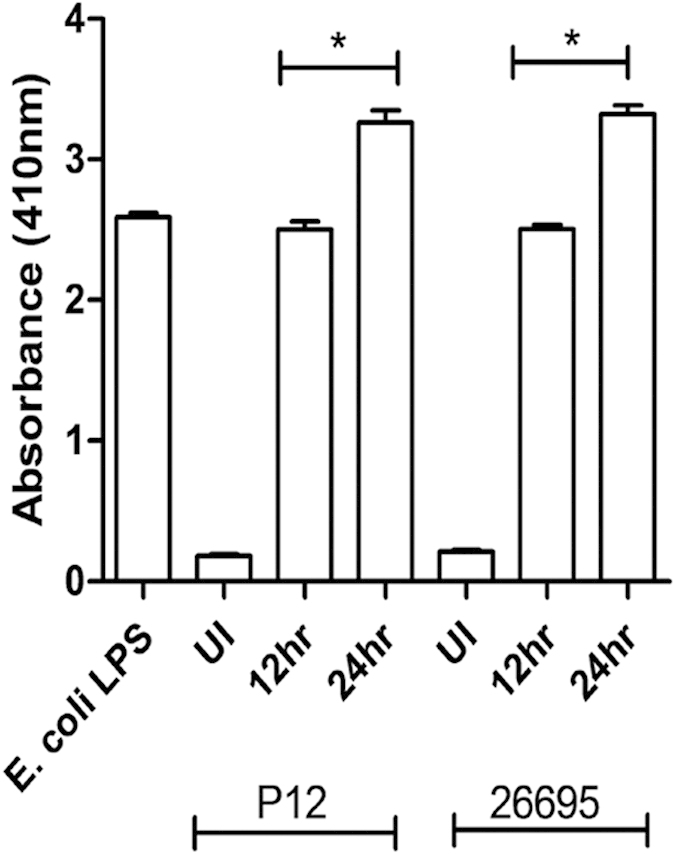
LPS determination. Shed LPS was determined in the culture supernatant from both directly and indirectly infected THP-1 cells with P12 and 26695 strains at the indicated time intervals. LPS was detected by Limulus amebocyte lysate assay (LAL) kit (Pierce Themo Scientific) as per the manufacture’s instruction and *E. coli* LPS served as a positive control. Data represent the mean and SD values of three replicates. UI indicates uninduced cells and * indicates significant difference where P < 0.05.

We next investigated if LPS shed by *H. pylori* could pass through permeable filter supports, and were responsible for Mincle expression. Cell culture supernatants were collected from both directly and indirectly infected THP-1 cells from 12 h and 24 h time points and the presence of LPS was determined. We could observe a positive signal for LPS in the culture supernatant of indirectly infected macrophages as compared to the non-infected macrophages for both the time points (Fig. 4). Indeed, our results also demonstrated that there was significantly more shedding of LPS in culture supernatant at 24 h as compared with the culture supernatant obtained at 12 h. This observation corroborated with our earlier observation illustrating increased expression of Mincle in a time dependent manner.

### Molecular docking studies indicated the possible Mincle interactions with Lewis antigens of *H. pylori* LPS

**Figure 5:**
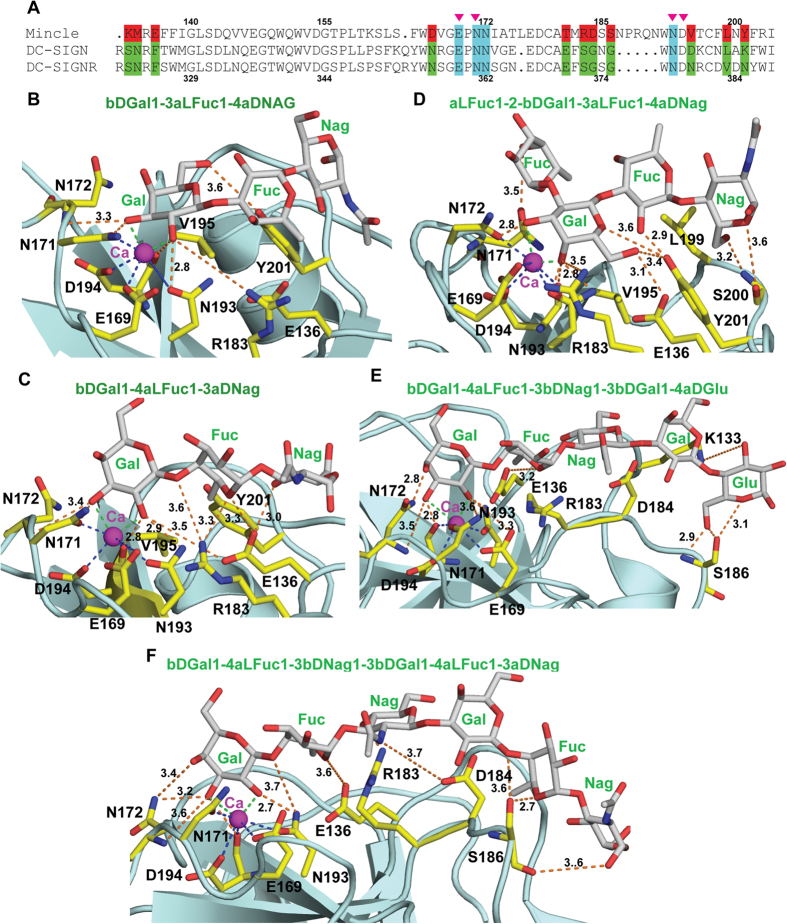
The proposed Mincle-oligosaccharide complex. Theoretical model of Mincle complexed with oligosaccharides found in *H. pylori*. Sequence of carbohydrates with glycosidic bond between the monomers is indicated on each panel. Protein and oligosaccharides are represented in ribbon (pale cyan color) and stick (grey atomic color) respectively. Mincle residues, co-coordinating Ca^2+^ and involved in possible interactions with the oligosaccharide, are represented in the yellow atomic color. Carbohydrate and protein mediated interactions with Ca ion are represented in dashed lines in green and blue color respectively. Ca^2+^ coordination geometry and coordination bond distance (2.35 to 2.6 Å) are maintained. Intermolecular interactions between the oligosaccharide and protein are depicted in orange colored dashed lines, and labeled distances are in Å. (**a**) Multiple sequence alignment of region of Mincle (PDB ID: 3WH2) with DC-SIGN (PDB ID: 1K9I) and DC-SIGNR (PDB ID: 1K9J) involved carbohydrate recognition and Ca^2+^ coordination. Sequence numbering of Mincle and DC-SIGNR are indicated above and below the sequence respectively. Magenta triangles designate the invariant residues involved in Ca^2+^ coordination. Residues highlighted in red and green background color corresponds to the variant residues in Mincle and DC-SIGN-R involved in ligand recognition. Invariant residues involved in substrate recognition are represented in cyan background color. (**b**) The theoretical model shows that 2′ and 3′ OH group of galactose (Gal1) are coordinating with Ca^2+^. Y201 and V195 form hydrophobic contacts with the saccharide. Both side and main chains of protein residues are involved in network of interaction with carbohydrate. (**c**) Mode of recognition of oligosaccharide is similar to one shown in B. (**d**) 3′ and 4′ OH groups of Gal2 are involved in oligosaccharide recognition by protein residues through Ca^2+^ coordination. Carbohydrate is docked on protein through hydrophobic interactions with Y201 and L199. (**e**) Ca^2+^ of protein molecule coordinates with 2′ and 3′ OH group of Gal1 of penta-saccharide. Aliphatic region of side chain of residues K133, E136, R183 and D184 interacts with penta-saccharide through hydrophobic interactions. (**f**) Recognition of hexa-saccharide through network of hydrogen and hydrophobic interactions and, ‘Ca^2+^’ mediated coordination.

Next we investigated whether *H. pylori* targets Mincle by using its LPS, and whether this binding occurs *via* Lewis antigenic determinants. Therefore, we investigated by docking whether Fucose containing Lewis ligands such as Lewis^X^ [(Galb134[Fuca133]GlcNAc)], Lewis^a^ [(Galb133[Fuca134]GlcNAc)], Lewis^b^ [(Fuca132Galb133[Fuca134]GlcNAc)], antidimeric X [(Galb134[Fuca133]GlcNAcb133Galb134[Fuca133]GlcNAc)] and human CD15 [(Galb134[Fuca133]GlcNAcb133Galb134Glc)] which are present in *H. pylori* LPS could accommodate in the ligand binding pocket of Mincle without steric clashes. Multiple sequence alignments of Mincle, DC-SIGN and DC-SIGNR revealed that residues recognizing saccharide moiety are invariant in SIGN and SIGN-R proteins whereas they were significantly different in the case of Mincle. Three polar or hydrophobic residues observed in DC-SIGN and DC-SIGNR were substituted to negatively charged residues in Mincle (Fig. 5a). Therefore, surface groove of Mincle that binds to ligand has negative potential. However, residues involved in coordination of Ca^2+^ were conserved among these proteins. Based on this, we speculate that Mincle might have evolved to recognize different saccharides or glycolipids or Lewis antigens as outlined in Fig. 5b–f.

Though tri-saccharides (Gal-Fuc-Nac) given in Fig. 5b and c have different stereochemistry, they fitted well in the ligand binding surface of the Mincle. As predicted, 3 and 4 hydroxyl groups of galactose (Gal1) of the saccharide can coordinate the Ca^2+^ of the protein molecule in case of both the tri-saccharides. This primary Ca^2+^ coordinated interaction may lead to alignment of saccharide molecule on the groove of Mincle. Hydrophobic side chain of the V195 interacts with apolar face of the galactose in both ligands (5B and 5C), whereas, N-acetyl glucosamine (NAG or GlcNAc) was recognized differently by the Mincle due to the structural differences in tri-saccharides. Tri-saccharide shown in Fig. 5b, a polar surface of the NAG has an alignment with the aromatic side chain of Y201 with hydrophobic interactions. In contrast, NAG in Fig. 5c has hydrophobic contacts with aliphatic region of the E136 side chain. In both (Fig. 5b,c) the cases, variant R183 has hydrogen bond interactions with the hydroxyl group of sugar moiety. Similarly, variant E136 forms hydrogen bond interaction with the hydroxyl group of the sugar as shown in Fig. 5b,c.

In case of tetra-saccharide (Fuc-Gal-Fuc-NAG) shown in Fig. 5d, saccharide can align on the groove of the binding surface only when 3^rd^ and 4^th^ hydroxyl groups of Gal2 coordinate with the Ca^2+^. Side chain of V195 has hydrophobic interactions with the Gal2 whereas L199 could interact with fucose (Fuc3) and NAG4 monomers. Side chains of R183, E136 and Y201 residues form intermolecular hydrogen bond or effect polar interactions with the hydroxyl group of saccharides.

Comparison of ligand recognition surface of DC-SIGNR with Mincle revealed that Mincle has lengthier surface groove. Therefore, Mincle could recognize the longer oligosaccharides such as penta- and hexa-saccharides. We docked both penta (Gal-Fuc-NAG-Gal-Glu) and hexa (Gal-Fuc-NAG-Gal-Fuc-NAG)-saccharides on the surface groove of Mincle and both fit well in the binding surface. In both the cases, Gal1 of the ligand is recognized through Ca^2+^ mediated coordination. Side chains of residues on the surface involved in both hydrophobic and hydrogen bond interactions with the ligand. Terminal glucose (Glu) and NAG of penta- and hexa- saccharides are recognized by the main chain groups of S186. Recognition of first and terminal residues of ligand could help in in-line alignment of the saccharide on the ligand binding groove that could reinforce the network of secondary interactions between internal sugars and protein molecule. Our results showed that, binding of *H. pylori* LPS to Mincle might be mediated by fucose containing Lewis antigens. Comparative structural analyses demonstrated that Mincle’s sugar binding surface is significantly different from DC-SIGNR and thus could recognize diverse linear saccharides.

### Mincle drives anti-inflammatory responses upon *H. pylori* infection

**Figure 6:**
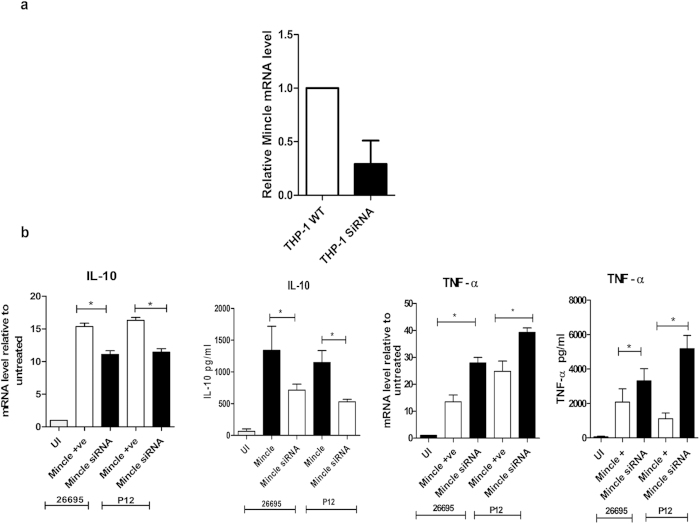
ssss(**a**) siRNA mediated knockdown of Mincle in THP-1 cells: qRT-PCR illustrated reduced transcript levels of Mincle after siRNA mediated knockdown (THP-1 SiRNA).Scrambled siRNA was used as control. 25picomoles of each siRNA was used. Mincle transcript levels were measured after 48 h of transfection. The transcript levels of Mincle were normalized to PPIB transcript level. (**b**) Mincle knockdown increases TNF-α production and decreases IL-10 production: Wild type (Mincle + ve, Mincle +  and Mincle) and Mincle silenced (Mincle siRNA) THP-1 cells were infected with *H. pylori* strains 26695 and P12 at an MOI of 50 for 12h and RNA was isolated and cell culture supernatant was collected. mRNA levels were quantified by qRT-PCR and the concentration of TNF-α and IL-10 secreted into the culture supernatant was determined by ELISA. Statistical significance was determined by student’s T test where P < 0.05 (*) was considered as significant.

To further examine the functional significance of the increased levels of Mincle mRNA in infected THP-1 cells, we used siRNA mediated gene silencing approach to knockdown the Mincle gene in THP-1 cells and confirmed the same by qRT-PCR. The expression of Mincle mRNA was down regulated by 80% in Mincle silenced THP-1 cells (Fig. 6a). We also determined the role of Mincle receptors in *H. pylori* mediated innate immune functions. Interestingly, our results demonstrated a significant upregulation and secretion of TNF-α in Mincle silenced (knockdown) THP-1 cells as compared to the wild type THP-1 cells. The amount of IL-10 expressed and secreted by Mincle silenced THP-1 cells was also evaluated in comparison to the THP-1 wild type cells. Our results show that IL-10 induction was decreased significantly in Mincle silenced THP-1 cells relative to the wild type THP-1 cells (Fig. 6b).

Collectively, our results suggest that Mincle expression occurs as an innate immune response towards *H. pylori* infection and it likely plays a crucial role in homeostasis of cytokine mediated pro and anti-inflammatory responses.

## Discussion

Over the past few years, many studies described C-type lectins (CLRs) as primary mediators of diverse immune interactions, most notably in the recognition of various pathogens and host antigenic determinants^39^. However, their role in *H. pylori* infection has not been completely known. DC-SIGN is one of the CLRs whose activation has shown to be crucial for *H. pylori* infection^40^. Binding of *H. pylori* to DC-SIGN blocks maturation of naive T cells to Th1 cells; this prevents *H. pylori* clearance by host immune system^40^. Gringhuis *et al.* have also reported that DC-SIGN interacts with Lewis antigens, and modulates the cytokine expression^40^. With this background, we attempted to identify additional CLRs linked to *H. pylori*. The present study successfully deciphered the role of yet another CLR, Mincle, in mediating innate responses during *H. pylori* infection. The CLRs are made of transmembrane proteins with a characteristic carbohydrate recognition domain (CRD)^41^ composed of two protein loops and two anti-parallel beta-sheets, made stable by highly conserved disulfide bonds plus four calcium binding sites. Owing to this arrangement, binding of ligands by CLRs is mostly a calcium dependent process^41^,^42^. The cytoplasmic domains of CLRs are frequently characterized as immune-receptor tyrosine-based activation motif (ITAM)-bearing adaptors such as Fc-receptor common γ chain (FcRγ)^43^. Some C- type lectin receptors such as Dectin-1 and Mincle directly recognize beta glucans on the surfaces of fungi and mycobacterial glycolipid trehalose dimycolate (TDM), respectively^32^. The expression of Mincle gets triggered upon the onset of *Mycobacterium tuberculosis, Streptococcus pneumoniae, Candidia albicans* and *Malassezia* infections^42^,^44^,^45^; this corroborates with our observations wherein Mincle expression was upregulated upon *H. pylori* infection (Fig. 1).

Our study revealed that Mincle mRNA transcript levels and surface expression of Mincle on PMA differentiated THP-1 cells were potentially triggered upon encounter with *H. pylori* in a time dependent manner. This strongly suggests an active involvement of Mincle in recognizing *H. pylori* during the course of infection. This is perhaps the first effort to elucidate the role of Mincle in *H. pylori* associated inflammation. We also found that the heat killed *H. pylori* retained stimulatory capability and was recognized by Mincle, ruling out the possibility of the involvement of any intact protein in recognition of *H. pylori*. Similar findings have also been reported by Yamasaki *et al.* wherein they demonstrated the ability of heat killed pathogenic fungus *Malassezia* to induce NFAT-GFP activation in Mincle reporter cells^31^. Considering the ability of both the live and heat killed *H. pylori* to induce Mincle, it is interesting to ascertain if the enhanced expression of Mincle was dependent purely on the interaction or any direct/indirect effect of host cell contact. In this context, our data indicated that Mincle expression was independent of host cell contact and there was no significant difference in Mincle expression either in directly or in indirectly infected THP-1 cells (Fig. 3). One possible explanation for this observation could be due to gastric epithelial cells secreting IL-8 when in contact with *H. pylori*, to recruit monocytes and macrophages to the gastric mucosa^33^. The peripheral inflammatory environment during chronic *H. pylori* infection might be dominated by macrophages where *H. pylori* LPS interacts with Mincle. Further, mononuclear cell infiltration in the lamina propria is seen as a cardinal feature of *H. pylori* induced chronic infection which has also been demonstrated at the level of gastric tissue samples of infected patients^46^,^47^. Similarly, we observed the expression of TNF-α and IL-10 by THP-1 cells in a contact independent manner (Fig. 3c). Our observations also confirm that the production of pro- and anti-inflammatory cytokines is not regulated at the host cell contact and it is more of a generic response towards the pathogen. Indeed, LPS is known to be a common trigger of innate immune responses^48^, and it is shown to be released by different Gram negative bacteria during both *in vitro* and *in vivo* growth^38^,^49^.

We also observed similar results wherein *H. pylori* releases LPS during infection that could pass through the permeable filter supports as detected by the LAL test (Fig. 4). Our results showed that the LPS released from *H. pylori* increases in a time dependent manner, which is essentially in agreement with the observation that Mincle expression also increased over time. Given these observations, it is clear that release of the LPS during *H. pylori* infection might be responsible for Mincle activation and consequent pro- and anti- inflammatory cytokine production.

Ever since the discovery of Lewis antigens in the LPS of *H. pylori*, a number of different biological functions such as increase in colonization, avoidance of host recognition, immune cells modulation and triggering of gastric autoimmunity have been associated with Lewis antigens^20^,^50^. Various Lewis antigens such as Lewis ^a^, Lewis ^b^, Lewis ^x^ and Lewis ^y^ are known ligands for DC-SIGN^40^,^51^,^52^. Moreover, dendritic cells and macrophages are the main targets for LPS^53^. Therefore, it is possible to surmise that the Lewis antigens might also mediate LPS binding to Mincle. Our hypothesis of LPS binding to Mincle through Lewis antigens is supported by the docking studies (Fig. 5). All the Lewis antigens outlined in Fig. 5 were fitted well with optimum network of interactions and minimum steric clashes and that indicated the possible interaction of Lewis antigens of *H. pylori* LPS with Mincle. Our observations in this context supported the interaction of Lewis ^x^, Lewis ^a^, Lewis ^b^ and human CD15 with Mincle.

We also found that Mincle silenced THP-1 cells enhanced production of pro-inflammatory cytokine, TNF-α and abrogated the production of anti- inflammatory cytokine IL-10 when compared with wild type macrophages. Recently, it has been reported that DC-SIGN which recognized the fucose ligands of *H. pylori* could also down regulate the pro-inflammatory signaling pathway^40^. Conversely, our results do not align with the observations reported with pathogens such as *Mycobacterium* and HIV which express mannosylated ligands and upregulate pro-inflammatory signaling pathways^54^,^55^. It has been demonstrated that Mincle induction on macrophages in response to BCG increases the production of TNF-α and MIP-2 *in vitro*^55^. This disparity could be due to a very different composition of capsular antigens in *H. pylori*.

Given that most of the virulence genes of *H. pylori* encode pro-inflammatory functions, upregulation of Mincle seems to be one of the possible mechanism by which *H. pylori* obliterates excessive pro-inflammatory cytokine secretion by IL-10 induction and may dampen inflammation in its own favor. In view of this possibility, our study suggests that other than the TLRs, Mincle induced anti-inflammatory cytokine production may contribute to maintenance of chronic persistence of *H. pylori*. It also provides an insight on differential role of fucosylated and mannosylated Mincle ligands in altering innate immunity to specific pathogens. However, to further decipher the role of Mincle in host defense or *H. pylori* survival, *in-vivo* mouse models need to be established.

In conclusion, we propose an interaction of *H. pylori* LPS and its released form with Mincle. We earlier hypothesized that during infection, shed LPS cross the epithelial lining and reach lamina propria as well as the endothelial layer of inflamed gastric mucosa and consequently increase the secretion of IL-6, IL-8 and IL-1β, which in turn attract the monocytes and macrophages to the site of infection. At this stage, Mincle might interact with Lewis antigens of LPS and lead to the production of IL-10. Therefore, upregulation of Mincle by LPS of *H. pylori* possibly fine tunes adaptation of *H. pylori* strains to their individual hosts; this might facilitate avoidance of detrimental host responses thus contributing crucially during chronic *H. pylori* infection.

Given that *H. pylori* eradication has become uncertain due to emergence of increased antimicrobial resistance^56^, the development of newer interventions such as immunotherapeutics and vaccines has become inevitable; this requires a comprehensive understanding of host-pathogen interactions. A systematic unraveling of host mediators of *H. pylori*-induced pathogenesis could possibly identify potential drug targets for therapeutic intervention against *H. pylori*-associated disease. With the understanding of the innate immune modulation triggered by *H. pylori* and the interplay of pro-inflammatory and anti-inflammatory signals, the immune response could be fine-tuned therapeutically to successfully eradicate the bacterium. Our observations therefore constitute important co-ordinates of innate immune functions mediated by *H. pylori* and therefore could be helpful in strengthening immune based control and eradication strategies.

## Materials and Methods

### *H. pylori* culture

*H. pylori* strains P12 and 26695 were routinely maintained on GC agar (BD Difco, USA) plates with 10% heat inactivated horse serum (Invitrogen) and 1% vitamin mixture (Sigma) supplemented with vancomycin (10 mg/ml), nystatin (2 mg/ml) and trimethoprim (2.5 mg/ml) (Himedia) under microaerophilic conditions (10% CO_2_ and 5% O_2_) at 37 °C for 48 h. *H. pylori* were killed by heating at 98 °C for 20 min.

### Macrophages stimulation

The human monocyte/macrophage THP-1 cells were procured from National Centre for Cell Sciences, Pune, India. The cells were cultured in RPMI 1640 medium, (Invitrogen life technologies) with 10% (v/v) heat inactivated fetal bovine serum (FBS) (Invitrogen Life technologies), 1% anti-biotic and anti-mycotic solution and were maintained at 37 °C with 5% CO_2_. The THP-1 cells were differentiated into macrophages, upon treatment with 10 ng/mL phorbolmyristate acetate (PMA) (Sigma) for 48 h. After differentiation the culture media was aspirated and replaced by fresh media with 10% FBS and were further maintained for another 24 h.

### Mincle knockdown with siRNA

siRNA against the Mincle (on target plus SMART oligonucleotides) containing four isoforms of the mincle gene was synthesized from Dharmacon (GE Healthcare). Scrambled siRNA was used as negative control while siRNA against cyclophilin was used as a positive control. PMA treated THP-1 cells (2 × 10^5^) were seeded into 24 well plate and were maintained in serum and antibiotic free RPMI 1640 medium for transfection. THP-1 cells were transfected with 3 μl of 10 μM stock of target plus SMART mincle siRNA using lipofectamine LTX PLUS (Invitrogen, Life technologies) according to the manufacturer’s instructions. Five hours post transfection, the medium was replaced with fresh RPMI 1640 medium containing 10% FBS and 1X antibiotic-antimycotic. For knockdown of Mincle at protein level, THP-1 cells were maintained for another 72 h.

### Infection assay

*H. pylori* strains 26695 and P12 were harvested from GC agar plates in 1X phosphate buffer saline (PBS) (Invitrogen Life technologies). Bacteria were pelleted down by centrifugation at 4000 rpm for 5 min and were washed twice with 1X PBS. THP-1 cells were infected with strains 26695 and P12 at multiplicity of infection (MOI) of 50 in serum and antibiotic free RPMI 1640 medium for 24 h. For indirect infection assay, six well Transwell permeable support plate (Corning) containing 0.4-μm pores was used. Cells were grown in lower compartment of the transwell permeable plate, and upper permeable filter units were kept in 6 well microplate and were placed in RPMI containing 10% FBS for 12 h at 37 °C before infection. *H. pylori* strains 26695 and P12 were harvested from GC agar plate in 1X PBS and infection was carried out at an MOI of 50, as described above. *H. pylori* strains were inoculated in the upper compartment of the permeable transwell unit and were incubated at 37 °C under 5% CO_2_ for another 24 h.

### ELISA for IL-10 and TNF-α

The level of secreted TNF-α, and IL-10 were measured in supernatants collected post infection. The cytokine levels were quantified using sandwich based ELISA kit (e Biosciences), as per the manufacturer’s instructions. Standard curve for the cytokines were obtained by using the recombinant proteins provided in the kit.

### Estimation and analysis of Mincle expression: RNA isolation, qRT-PCR and Flow cytometry

Total RNA was extracted by using Trizol (sigma), and 1 μg of purified RNA was treated with DNase (sigma) and reverse transcribed by using first strand synthesis system (Invitrogen Life technologies) as per the manufacturer’s instructions. qRT PCR was carried out by using Eppendorf real time machine by utilizing the primers as given in Table 1. Briefly, the reaction was performed in 10 μl volume containing 5 μl of SYBR green (Bioline), 0.2 μl forward primer and 0.2 μl reverse primer, 40 ng c-DNA and remaining amount of DNase-RNase free water (Invitrogen, Life technologies). Real time PCR was performed as follows for all Mincle, TNF-α and PPIB 95 °C for 10 min, 95 °C for 15 sec, 58 °C for 15 sec, and 72 °C for 15 sec. Mean fold changes were analyzed by ∆∆CT method as described earlier^57^. To determine the surface expression of mincle, PMA differentiated THP-1 macrophages were incubated with 10 μg/ml mouse anti-mincle mAb or isotype matched control IgG2b antibody for 60 min at 4 °C followed by incubation with FITC-conjugated goat anti mouse IgG (sigma) for another 45 min at 4 °C . Cells were washed and resuspended in 1X PBS with 1% BSA. The fluorescence was measured by BD-FACS Canto II and results were analyzed by Flowjo software.

### Computational modeling of Mincle interaction with Lewis antigens

Oligosaccharides used for docking were modeled and energy minimized using, GLYCAM, a web based server (http://glycam.org/). DC-SIGNR (PDB ID: 1K9J) and DC-SIGN (PDB ID: 1K9I) complexed with GlcNAc2Man are used as template for manual docking of different oligosaccharides on Mincle. Ligand free structure of Mincle is superposed on DC-SIGNR and DC-SIGN using the COOT program^58^ GlcNAc2Man saccharide, present in the complex structures, was used as reference for determining the orientation of different oligosaccharides outlined in the Fig. 5B–F. Following criteria were used in manual docking of the oligosaccharide: OH groups of sugar of one of the monomers should be positioned in such an orientation that could able to coordinate with Ca^2+^. Ca^2+^ coordination with OH groups should lead to an in-line alignment of sugar moieties on the surface groove of the Mincle. Alignment of saccharide should have minimum short contacts and optimum interactions with residues of the protein molecule. We carried out validation for steric clashes between oligosaccharides and the Mincle using the MolProbity server (http://molprobity.biochem.duke.edu/)^59^. Overall, interaction of oligosaccharides to Mincle was found to be highly similar to the template DC-SIGNR-GlcNAc2Man (PDB ID: 1K9J) and DC-SIGN-GlcNAc2Man (PDB ID: 1K9I) complex structures.

### Lipopolysaccharide (LPS) estimation

Release of *H. pylori* LPS during direct and indirect infection in THP-1 cells was measured in culture supernatant at different time intervals. The level of shed LPS was determined by Limulus amebocyte lysate assay (LAL) kit (Pierce Themo Scientific) according to the manufacture’s instruction. 50 μl of cell culture supernatant was added in triplicate to 50 μl of LAL in a pyrogen-free microtiter plate. The mixture was incubated at 37 °C for 10 min, and 100μlof chromogenic substrate solution was added and color development was terminated by addition of 20% acetic acid. The optical density was measured at 410 nm.

### Statistical Analysis

Statistical calculations were performed by using GraphPad Prism 5 software. For ELISA and qRT PCR, statistical evaluation was performed by using student’s *t*-test and one way ANOVA and p ≥ 0.05 was considered non-significant.

## Additional Information

**How to cite this article**: Devi, S. *et al.* Induction of Mincle by *Helicobacter pylori* and consequent anti-inflammatory signaling denote a bacterial survival strategy. *Sci. Rep.* **5**, 15049; doi: 10.1038/srep15049 (2015).

## Figures and Tables

**Table 1:** List of qRT-PCR primers.

S. No	Gene name	Primer sequences	Reference
1.	Mincle	F-5′- ACA CCA TTT CCT GGG CGT TA-3'	This study
		R-5′-TTT GTC AAA GGT GTG CCG TC-3'	
2.	Peptidylpropyl isomerase B (PPIB)	F-5′-ATG TAG GCC GGG TGA TCT TT-3'	This study
		R-5′-TGA AGT TCT CAT CGG GGA AG-3'	
3.	TNF-α	F-5′-TTC TCC TTC CTG ATC GTG GC-3'	This study
		R- 5′-ACT CGG GGT TCG AGA AGA TG-3'	
4.	IL-10	F- 5′ AGAACCTGAAGACCCTCAGGC3′	60
		R- 5′ CCACGGCCTTGCTCTTGTT 3′	
